# Impact of *Ruminococcus torques* Administration on Glucose Tolerance and Hepatic Selenoprotein Expression in Selenium-deficient Mature Female Mice

**DOI:** 10.1007/s12011-026-05106-5

**Published:** 2026-05-13

**Authors:** Ying-Chen Huang, Wen-Hsing Cheng

**Affiliations:** 1 Department of Food Science, Nutrition and Health Promotion, Mississippi State University, Mississippi State, MS 39762 USA; 2 Department of Nutrition and Food Sciences, Texas Woman’s University, Denton, TX 76209 USA

**Keywords:** Selenium deficiency, Glucose intolerance, Insulin resistance, Gut microbiota, *Ruminococcus torques*

## Abstract

We previously found that dietary selenium (Se) deficiency and age increase the fecal abundance of *Lachnospiraceae* in aged telomere-humanized mice in a sexually dimorphic manner. Although *Lachnospiraceae* are key contributors of short-chain fatty acids to the host, different taxa within this family exert distinct effects on host physiology. Among them, *Ruminococcus torques* has been associated with type 2 diabetes. In the present study, we aimed to determine whether, and how, *R. torques* interacts with dietary Se to influence type 2 diabetes-like symptoms. Sixteen weaning female C57BL/6J mice were fed either a Se-adequate or Se-deficient diet for 26 weeks. From weeks 21 to 25, half of the mice in each dietary group received daily oral gavage of *R. torques* (2 × 10⁸ CFU in 0.2 mL). All mice were euthanized at week 26. Dietary Se deficiency induced glucose intolerance (13%) and insulin resistance (16%) (*P* < 0.05). While *R. torques* administration was associated with a reduction in glucose levels at 30 min post-injection in Se-deficient mice, it did not significantly alter the overall glucose area under the curve, insulin resistance, or fasting glucose levels. Se deficiency reduced the relative abundance of *Lactobacillus spp*., *F. prausnitzii*, and *Roseburia spp./E. rectale* in the cecal content, and these taxa were unaffected by *R. torques* treatment. In contrast, Se deficiency increased the relative abundance of *R. torques* and *E. coli* in cecal samples, with *E. coli* levels further elevated by *R. torques* gavage. Notably, *R. torques* oral gavage *1*) decreased SELENOP and GPX1 protein levels in the liver, but not in skeletal muscle, of Se-adequate mice; *2)* increased liver GPX1 protein levels in Se-deficient mice. Altogether, *R. torques* administration exerts a marginal effect on glucose excursion and increases both liver GPX1 protein levels and cecal *E. coli* abundance in Se-deficient mature female mice with diabetic symptoms.

**Supplementary Information:**

The online version contains supplementary material available at 10.1007/s12011-026-05106-5.

## Introduction

The *Lachnospiraceae* are a family of anaerobic bacteria within the *Clostridiales* order of the *Firmicutes* phylum, and include species previously classified as part of *Clostridium* cluster XIVa [1, 2]. This family is abundant and dominant in the unperturbed adult gut microbiota [3, 4]. However, only 25% of the genomes are closely related (> 97% similarity) to known species, and 28% do not match any records of existing databases [3]. Interestingly, 19% of sequences are closely related to recently isolated butyrate-producing bacteria from *Clostridium* clusters XIVa and IV, while 18% are related to *Ruminococcus obeum* and *Ruminococcus torques*, which are members of XIVa [3]. *Ruminococcus* is currently considered a polyphyletic genus, with species distributed across two distinct families: the *Ruminococcaceae* and the *Lachnospiraceae* [5]. Based on phenotypic traits, 16S rRNA gene sequence similarity [6], phylogenetic analysis, G + C content of genomic sequence, and DNA-DNA hybridization studies, the species *R. torques*, *R. obeum*, *R. lactaris*, *R. gnavus*, and *R. gauvreauii* are classified within the *Lachnospiraceae* family [7].

As a core component of the gut microbiota, *Lachnospiraceae* colonize the intestinal lumen from birth, exhibiting age-dependent increases in species richness and relative abundance. *Lachnospiraceae* have been implicated in obesity and diabetes in both humans and mouse models [8–14]; however, their specific role in type 2 diabetes remains unclear. We have previously shown that long-term dietary selenium (Se) deficiency induces type 2 diabetes-like symptoms in both male and female telomere-humanized mice [15], and increases the fecal abundance of *Lachnospiraceae* at 18 months of age in both sexes, but only in females at 24 months, based on 16S rRNA gene sequence analyses [16]. In contrast, *Akkermansia muciniphila*, a mucin-degrading bacterium that comprises 3%–5% of the microbial community in healthy individuals [17], shows the most pronounced age-associated enrichment in response to dietary Se deficiency, but only in male telomere-humanized mice. Within the *Lachnospiraceae* family, *R. torques* is another mucin-degrading bacterium whose abundance is positively associated with irritable bowel syndrome in humans [18]. Because oral gavage of *A. muciniphila* alleviates type 2 diabetes-like symptoms in Se-deficient male mice, these findings collectively suggest that mucin-degrading bacteria may play a critical role in modulating inflammatory responses at the gut mucosal surface [16]. Therefore, we aimed to investigate the effects of oral administration of *R. torques* on dietary Se deficiency-induced type 2 diabetes-like symptoms, as well as on symbiotic changes involving other mucin-degrading or short-chain fatty acid-producing bacteria in mature female mice.

## Materials and Methods

### Culture of *Ruminococcus torques*

*R. torques* (ATCC BAA-2281) were cultured under strict anaerobic conditions using the Anoxomat III Jar system (Advanced Instruments, Norwood, MA) in modified reinforced clostridial medium following ATCC protocol. Under anaerobic conditions, cultures were washed and concentrated in sterile PBS containing 25% (vol/vol) glycerol to a final concentration of 1$\:\times\:$10^10^ CFU/mL, followed by immediately freezing and storage at − 80 °C. Before oral administration, stocks were thawed and diluted anaerobically in sterile PBS with 2.5% glycerol to a final concentration of 1$\:\times\:$10^9^ CFU/mL.

### Mice, Diets, and Treatment

**Fig. 1:**
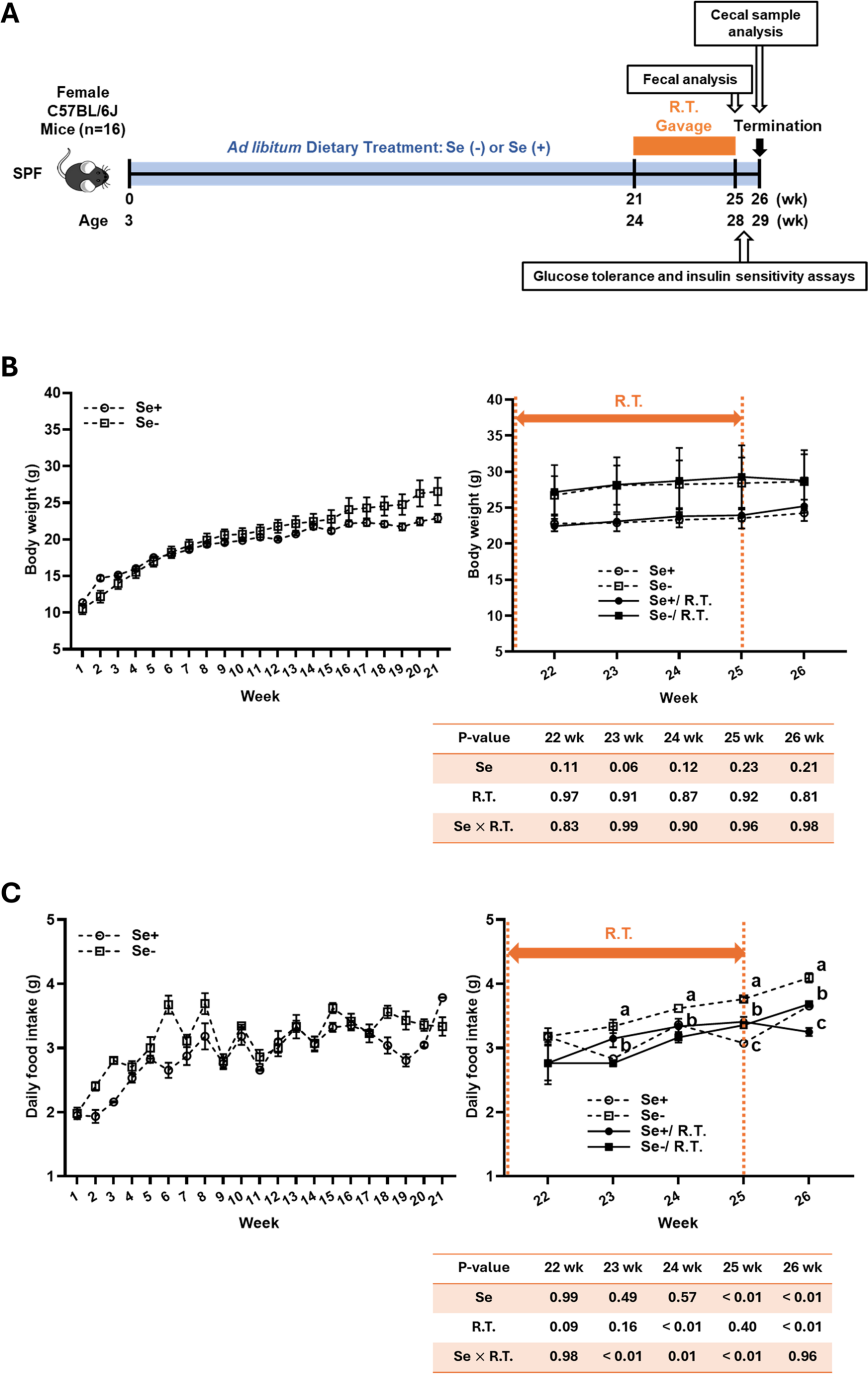
Experimental design, body weight, and food intake in female C57BL/6J mice. Schematic diagram illustrating experimental design (**A**), body weight (**B**), and food intake (**C**) in 3-weeks-old female C57BL/6J mice fed either a Se-adequate or Se-deficient diet for 26 weeks. Twenty-one weeks after the start of dietary manipulation, mice received daily oral gavage with *R. torques* or mock treatment for 4 weeks. Values are means ± SEMs (*n* = 4). Means without sharing a common letter at a given time point differ, *P* < 0.05. R.T., *R. torques*; Se+, selenium-adequate diet; Se−, selenium-deficient diet; SPF, specific pathogen-free

As shown in Fig. 1A, sixteen weaning female C57BL/6J mice were housed under conventional specific pathogen-free (SPF) conditions and fed either a Se-deficient or Se-adequate torula yeast-based purified diet for 26 weeks, as described previously [16]. At week 21, mice (*n* = 4 per group) received a daily oral gavage of either 2$\:\times\:$10^8^ CFU live *R. torques* or a control vehicle (2.5% glycerol in 200 µL) for 4 weeks. Mice were handled aseptically in a controlled environment with 12-hour light/dark cycle (lights off from 6 p.m. to 6 a.m.), with *ad libitum* access to food and water. Body weight and food intake were monitored weekly. Fresh fecal samples were collected before and after *R. torques* treatment, snap-frozen in liquid nitrogen, and stored at − 80 °C. Two days after the insulin sensitivity assay, mice were fasted for 6 h, anesthetized with carbon dioxide, and euthanized by cardiac exsanguination. Liver, skeletal muscle, and cecal contents were collected, rapidly frozen in liquid nitrogen, and stored at − 80 °C for further analyses. All procedures were approved by the Institutional Animal Care and Use Committee of Mississippi State University.

## Glucose Tolerance and Insulin Sensitivity

At weeks 21 and 26 of dietary intervention, following an 8-hour fast, mice were intraperitoneally injected with glucose (1 g/kg body weight) or insulin (0.25 U/kg body weight) (Sigma Aldrich, St. Louis, MO). Blood glucose concentrations were measured using a glucose meter (Bayer Contour Next EZ, Ascensia Diabetes Care US, Inc., Parsippany, NJ) from a drop of tail vein samples collected at 0 (baseline), 0.25, 0.5, 1, 1.5, and 2 h post-injection. Insulin sensitivity was assessed 2 days after glucose tolerance assays. The area under the curve (AUC) was calculated to quantify glucose and insulin responses.

### Bacterial Genomic DNA Extraction and Quantitative PCR (qPCR) Analysis

Bacterial DNA was extracted from cecal contents and feces using the QIAamp PowerFecal Pro DNA Kit (#51804, QIAGEN, Germantown, MD) following the manufacturer’s instructions. Universal primers targeting the V4 region of the bacterial 16S rRNA gene were used for amplification (primer sequences listed in Supplemental Table 1). qPCR was conducted on a QuantStudio 5 Real-Time PCR System (#A34322, Applied Biosystems) using PowerUp™ SYBR™ Green Master Mix (10 µL reactions; #A25741, Applied Biosystems, Waltham, MA) under the following thermal conditions: 95 °C for 2 min, then 40 cycles of 95 °C (5 s) and 60 °C (30 s). Total bacterial load in fecal samples was quantified using the ΔCT method. Due to normalization of ΔCT values by subtraction against the control group, two-way ANOVA was not appropriate for analyzing ΔΔCT data, as a designated control could not be applied across two factors. Instead, unpaired t-tests were used for statistical analysis.

## Immunoblotting

Tissues were homogenized in RIPA lysis buffer with protease inhibitors (# sc-24948, Santa Cruz Biotech, Dallas, TX) and centrifuged at 12,000 × g for 10 min at 4 °C. Supernatants (30 µg protein per lane) were separated by 14% SDS-PAGE and subsequently transferred to polyvinylidene difluoride membranes. Membranes were incubated overnight at 4 °C with primary antibodies (listed in Supplemental Table 2), followed by HRP-conjugated secondary antibodies for 2 h at room temperature. Signals were developed using Clarity Western ECL substrate, and images were captured and quantified using a Chemidoc-XS system with the volume tool in Image Lab Software (Bio-Rad Lab, Hercules, CA). Protein levels were normalized to albumin, β-tubulin, or total AKT. Uncropped images are shown in Supplemental Fig. 3.

### Statistical Analysis

Data are presented as means ± SEM. Datasets were analyzed by two-way ANOVA followed by Tukey’s post hoc test, except for qPCR-based assays, which were evaluated using unpaired *t*-tests. Statistical analyses were performed using SAS (version 9.4) and GraphPad Prism (version 8.0). A significance level of α = 0.05 was used for all tests.

## Results

### Body Weight and Food Intake

Weaning female C57BL/6J mice exhibited steady increases in body weight (103–153%; *P* < 0.05; Fig. 1B) and food intake (56–70%; *P* < 0.05; Fig. 1C) over the 21-week dietary intervention. In mice receiving control oral gavage, dietary Se deficiency increased food intake (8–18%; *P* < 0.05) but had no effect on body weight. Daily oral gavage with *R. torques* resulted in a deduction in food intake in Se-deficient mice at weeks 23–26 (10–17%; *P* < 0.05); however, body weight remained unaffected. In *R. torques*-gavaged mice, dietary Se deficiency increased food intake at week 26 (13%; *P* < 0.05) but did not alter body weight.

### Changes in the Abundance of Specific Genera by Dietary Se Deficiency and *R. torques* Oral Gavage in Mice

**Fig. 2:**
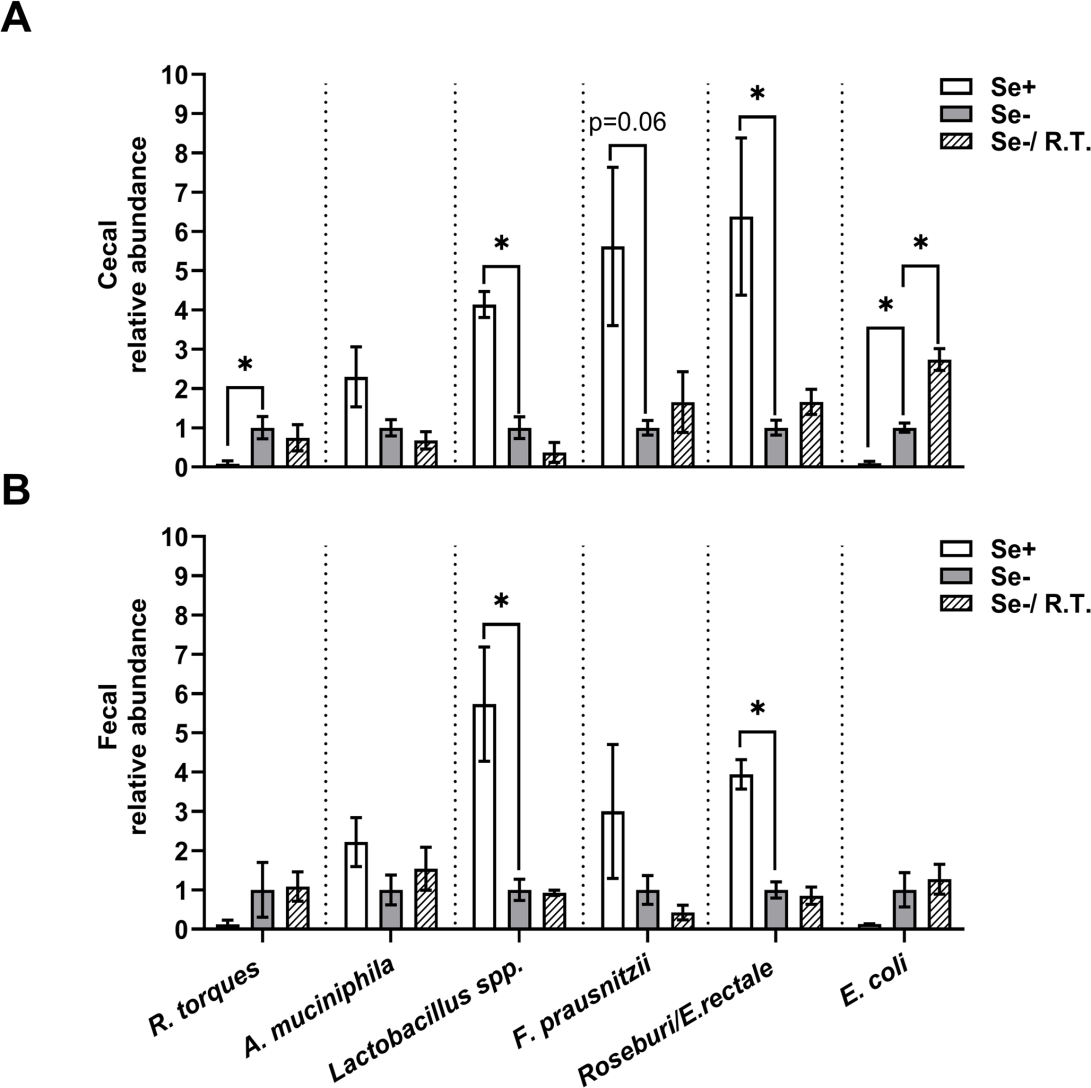
Cecal and fecal abundance of six bacteria taxa. Relative abundance of *R*. *torques*, *A*. *muciniphila*, *Lactobacillus spp*., *F*. *prausnitzii*, *Roseburia/E*. *rectale*, and* E*. *coli* in cecal (**A**) and fecal (**B**) samples from female C57BL/6J mice fed either a Se-adequate or Se-deficient diet. *R*. *torques* oral gavage was administered to Se-deficient mice (See Fig. 1A for detailed experimental design). Values are means ± SEMs (n = 4). *P < 0.05.R.T., *R*. *torques*; Se+, selenium-adequate diet; Se−, selenium-deficient diet

In Se-adequate mice, oral gavage with *R. torques* was associated with apparent increases (3- to 5-fold) in the abundances of *R. torques*, alongside decreases (52–82%; *P* < 0.05) in *Lactobacillus spp*. and *Roseburia /E. rectale* in the cecal and fecal contents (Supplemental Fig. 1). In mice receiving control oral gavage, dietary Se deficiency increased (*P* ≤ 0.06) the abundances of *R. torques* (10.9-fold) and *E. coli* (9.5-fold), while decreasing that of *Lactobacillus spp.* (3.1-fold), *F. prausnitzii* (4.6-fold), and *Roseburia /E. rectale* (5.3-fold) in cecal content (Fig. 2A). A similar trend was observed in fecal content (Fig. 2B), but only *Lactobacillus spp*. (4.7-fold) and *Roseburia /E. rectale* (2.9-fold) reached statistical significance (*P* < 0.05). In mice receiving oral gavage with *R. torques*, dietary Se deficiency increased the relative abundance of *E. coli* in both cecal and fecal contents (4- to10-fold; *P* < 0.05) and decreased that of fecal *Roseburia /E. rectale* by 54% (*P* < 0.05), but not in other taxa (supplemental Fig. 2).

### Marginal Impact of *R. torques* on Glucose Tolerance without Altering Insulin Sensitivity in Se-deficient Conventional Mice

**Fig. 3:**
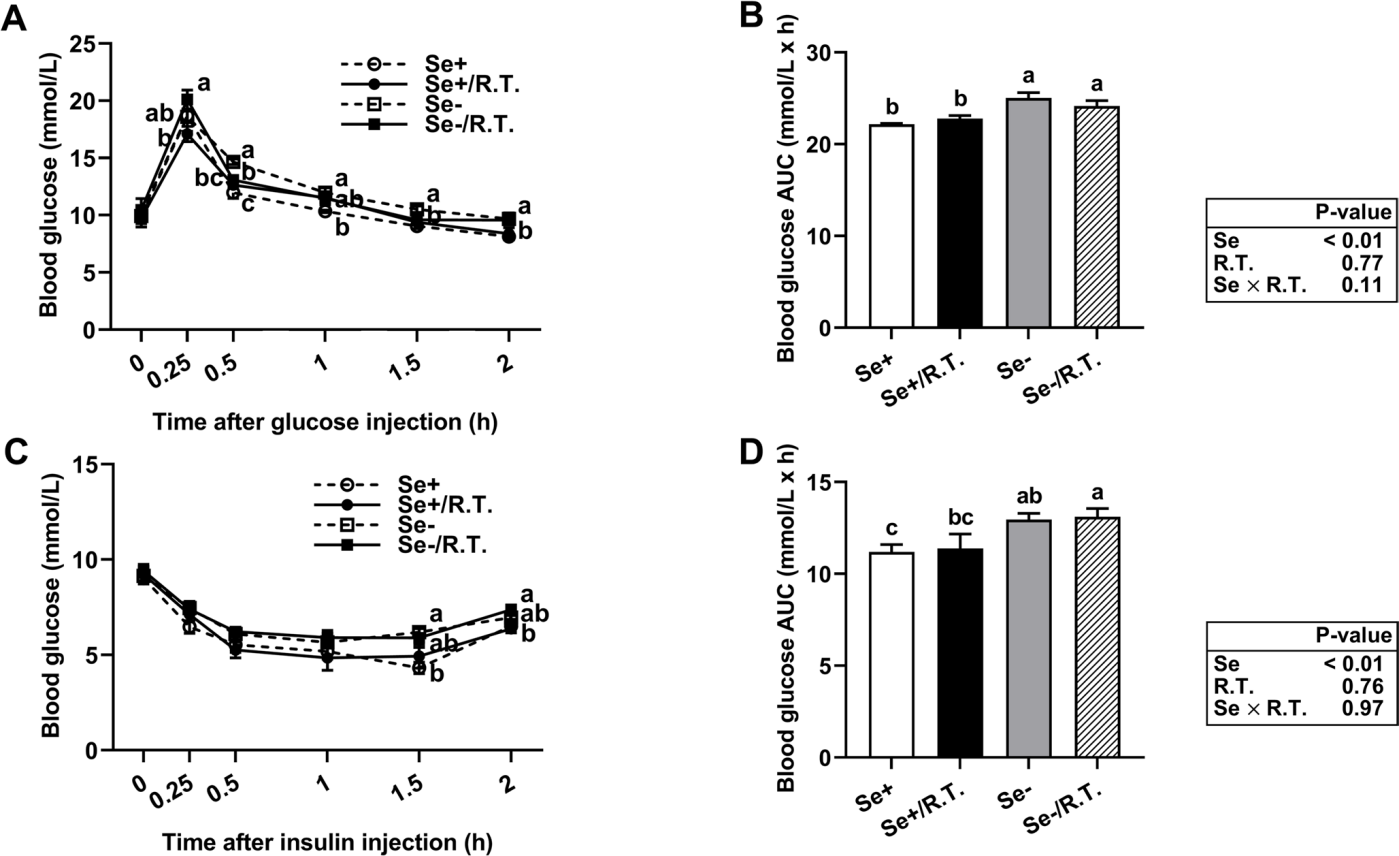
Glucose tolerance and insulin sensitivity assays in female C57BL/6J mice. Blood glucose levels were measured following intraperitoneal injection of glucose (1 g/kg; **A**, **B**) or insulin (0.25 U/kg; **C**, **D**) in female C57BL/6J mice fed either a Se-adequate or Se-deficient diet and given *R. torques* or mock oral gavage (see Fig. 1A for detailed experimental design). Mice were fasted 8 h prior to glucose or insulin injection. The average area under the curve was calculated from the data in panels A and C, with units expressed as mmol • h • L − 1 (B and D). Values are means ± SEMs (*n* = 4). Means without sharing a common letter at a given time point differ, *P* < 0.05. AUC, average area under the curve; R.T., *R. torques*; Se+, selenium-adequate diet; Se−, selenium-deficient diet

Se-deficient female mice at ~ 7 months of age exhibited glucose intolerance (Fig. 3A and B) and insulin resistance (Fig. 3C and D) over a 2-hour period following injections. However, fasting blood glucose concentrations were not affected by dietary Se deficiency or by *R. torques* oral gavage (Fig. 3A and C). While oral gavage with *R. torques* modestly alleviated (11% improvement; *P* < 0.05) Se deficiency-induced glucose intolerance at 0.5 h post-injection (Fig. 3A), it had no significant effect on overall glucose tolerance or insulin sensitivity across the entire time course in either the Se-deficient or Se-adequate mice.

### *R. torques* Oral Gavage and Dietary Se Deficiency Differentially affect Selenoprotein Expression in Muscle and Liver

**Fig. 4:**
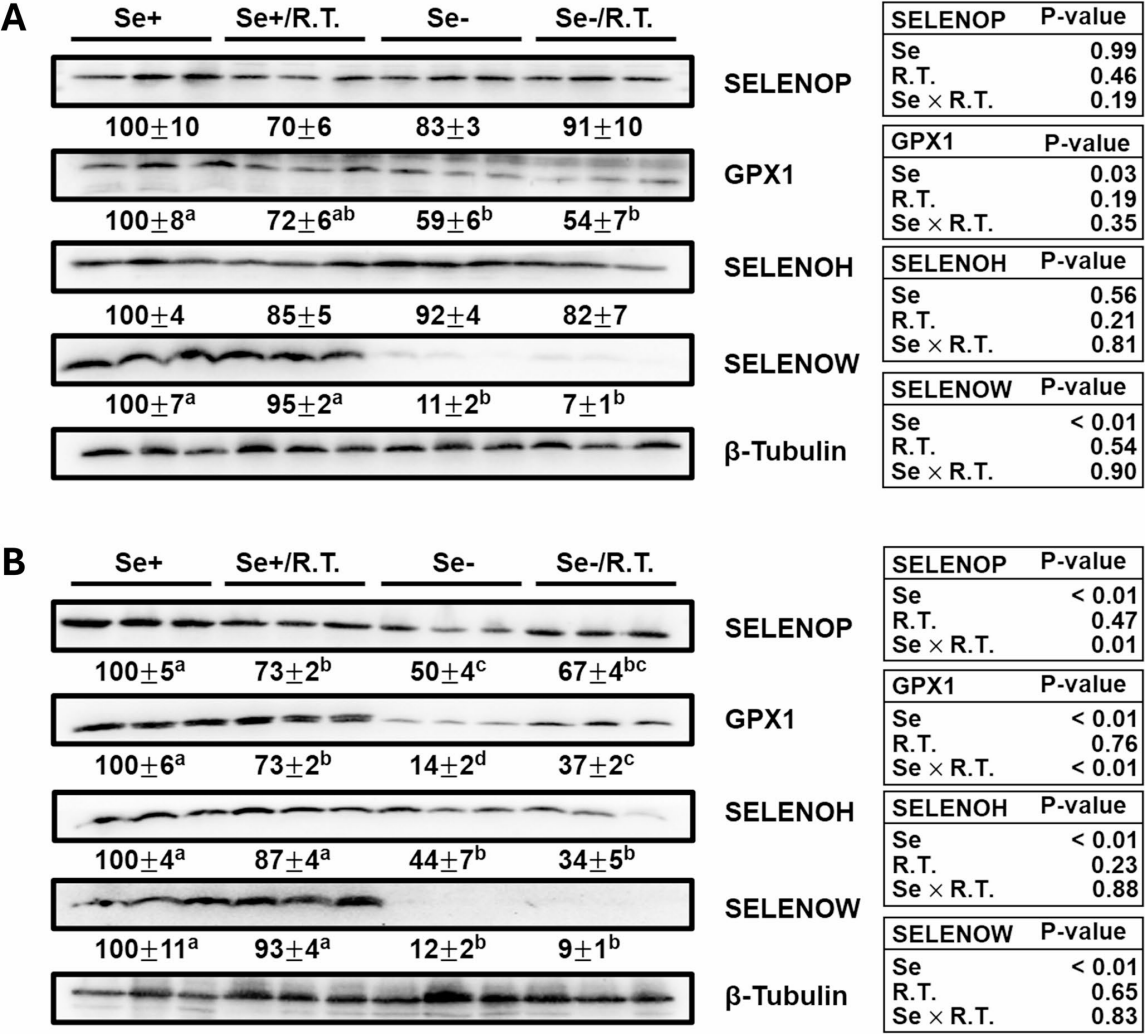
Western blot analysis of body Se status in female C57BL/6J mice. Selenoprotein expression in skeletal muscle (**A**) and liver (**B**) of female C57BL/6J mice fed either a Se-adequate or Se-deficient diet and given *R. torques* or mock oral gavage (see Fig. 1A for detailed experimental design). Protein levels were normalized to β-tubulin and expressed as a percentage of the Se-adequate control group. Values are means ± SEMs (*n* = 3). Means without sharing a common letter differ, *P* < 0.05. GPX1, glutathione peroxidase 1; R.T., *R. torques*; Se+, Se-adequate diet; Se−, Se-deficient diet; SELENOH, selenoprotein H; SELENOP, selenoprotein P; SELENOW, selenoprotein W

We next assessed body Se status in the liver and skeletal muscle by evaluating protein expression levels of selected selenoproteins central for systemic Se delivery (SELENOP) or sensitive to dietary Se deficiency (GPX1, SELENOH, and SELENOW). Western blot analysis revealed that dietary Se deficiency reduced (*P* < 0.05) the protein levels of GPX1 by 41% and SELENOW by 89% in muscle; whereas SELENOH and SELENOP expression remained unaffected (Fig. 4A). In the liver (Fig. 4B), Se deficiency decreased (*P* < 0.05) protein levels of SELENOP by 50%, GPX1 by 86%, SELENOH by 56%, and SELENOW by 88%. Notably, the reduction in hepatic GPX1 was partially reversed by *R. torques* oral gavage (1.6-fold increase; *P* < 0.05). In Se-adequate mice, oral gavage of *R. torques* reduced (*P* < 0.05) hepatic levels of both SELENOP and GPX1 by 27%, but had no effect on SELENOH or SELENOW in the liver, nor on any selenoprotein expression in muscle.

## Discussion

We have previously shown that long-term dietary Se deficiency induces type 2 diabetes-like symptoms in telomere-humanized mice aged 12 and 18 months in both sexes [15], as well as middle-aged and mature wild-type male mice [16, 19]. Here, compared to wild-type male mice aged 4–7 months [16], dietary Se deficiency in age-matched females induces glucose intolerance and insulin resistance to a lesser extent. Consistent with our findings, there is evidence that females are generally more insulin-sensitive than males [20, 21], and women have decreased susceptibility to fatty acid–induced peripheral insulin resistance [22]. In rodent models, males typically exhibit more pronounced diabetes symptoms than females [23–26]. These sex-related differences in type 2 diabetes-like symptoms may be partially attributed to the actions of estrogen and testosterone. For example, decreases in estrogen and increases in testosterone levels during menopause are associated with a loss of subcutaneous fat, a gain of visceral fat, and increased insulin resistance [27]. Indeed, we have previously shown that Se status under Se deficiency is tissue-specific and dependent on sex and age [28].

In contrast to the protective effects of *A. muciniphila* [16], oral gavage with *R. torques* results in only a marginal and transient reduction in Se deficiency-induced type 2 diabetes symptoms. An intriguing question arises: why do these two mucin-degrading bacteria have opposing impacts on Se deficiency-induced metabolic symptoms? The divergence likely stems from the delicate balance between beneficial mucus turnover and detrimental barrier degradation. While both are mucin-degrading bacteria, *A. muciniphila* facilitates mucosal homeostasis by stimulating host mucus production and generating short-chain fatty acids, such as acetate and propionate; these metabolites strengthen tight junctions and improve insulin sensitivity [16]. In contrast, *R. torques* appears to function more opportunistically. Its degradation of the protective mucus layer may occur independently of host homeostatic feedback mechanisms, potentially increasing the direct exposure of gastrointestinal epithelial cells to luminal pathogens and pro-inflammatory stimuli [29]. This pathogenic degradation is further evidenced by its association with inflammatory conditions like pasty vent in avian models and the elevated 16S rRNA gene counts observed in inflammatory bowel disorder, where the breakdown of endogenous mucus may inadvertently sustain the growth of non-mucolytic, pro-inflammatory bacteria [30, 31] Further, while *A. muciniphila* can symbiotically reverse mucosal barrier dysfunction and gut inflammation under Se-deficient conditions [16], the activity of *R. torques* is often associated with the release of oligosaccharides that fuel other potentially harmful bacteria like *E. coli* [31, 32]. Consequently, while *A. muciniphila* fosters a resilient, homeostatic environment, *R. torques* may compromise the gut barrier. This distinction explains why the impact of *R. torques* on Se deficiency-induced type 2 diabetes-like symptoms remains marginal and transient.

*R. torques* has been correlated with markers of insulin resistance in humans [33]. In our study, dietary Se deficiency enriched *R. torques* and induced type 2 diabetes-like symptoms in mice; however, whether this enrichment plays a causative role in the disease remains unknown. While oral administration of *R. torques* led to a modest reduction in blood glucose specifically at the 30-minute time point, it failed to significantly improve overall glucose AUC on insulin resistance in Se-deficiency female mice. We speculate that *(1)* female mice may exhibit greater baseline insulin sensitivity, potentially masking any subtle effects of *R. torques*; *(2)* interactions between *R. torques* and host selenoproteins may counterbalance each other’s influence on type-2 diabetes pathogenesis.

Although a recent study using the type strain *R. torques* ATCC 27,756 (BSL-1) demonstrated a pronounced alleviation of glucose intolerance in obese mice via oral gavage [2], we employed a non-type strain, *R. torques* ATCC BAA-2281 (BSL-2), and observed only a limited effect on Se deficiency-induced glucose intolerance. The functional divergence between these two strains likely stems from significant genomic and metabolic variations that influence their interaction with the host. This discrepancy may be attributed to the unique bioactive polypeptides produced by ATCC 27,756, which have been shown to directly modulate host metabolic pathways [2], a trait that may be absent or differentially expressed in the minimally characterized ATCC BAA-2281. We selected this strain because it is deposited in ATCC as a *Lachnospiraceae* bacterium. We have previously shown that dietary Se deficiency enriches the relative abundance of *Lachnospiraceae* in aged, telomere-humanized mice in a sexually dimorphic manner [16], suggesting that this specific lineage is highly sensitive to Se status. To better understand the differing efficacies of these two *R. torques* strains in modulating glucose excursions, future studies should consider factors such as host obesity status, strain-level genetic variation, and the underlying etiology of type 2 diabetes.

Other gut bacteria may respond to *R. torques* oral gavage and influence outcomes related to glucose intolerance and insulin resistance in Se-deficient mice. Indeed, Se deficiency in females decreases the abundance of *Lactobacillus spp.*, *F. prausnitzii*, and *Roseburia spp./E. rectale*, while increasing the abundance of *R. torques* and *E. coli* in the cecal content. However, *R. torques* oral gavage results in increased *E. coli* abundance in Se-deficient mice and decreased *Lactobacillus spp*. and *Roseburia spp./E. rectal* abundance in Se-adequate mice. We employed a targeted qPCR approach rather than broad 16S rRNA sequencing to quantify a specific panel of taxa (*Lactobacillus* spp., *F. prausnitzii*, *Roseburia spp.*/*E. rectale*, *R. torques*, and *E. coli*). These microbes were prioritized based on our previous sequencing data identifying them as key Se-responsive taxa [16]. This targeted strategy allowed us to compare the microbial responses elicited by *R. torques* gavage with those observed in our previous studies of *A. muciniphila* administration using the same quantitative metrics. Because these findings contrast with those observed following *A. muciniphila* oral gavage [16], these two mucin-degrading bacteria likely influence gut homeostasis and host health through distinct mechanisms.

These parallel shifts in the gut microbiota and host selenoproteins point to a context-dependent relationship between *R. torques*, Se status, and glucose metabolism. Dietary Se deficiency induced an expansion of potential pathobionts like *E. coli* and *R. torques*, together with a depletion of butyrate-producing *Roseburia*/*E. rectale*, a shift likely compromising gut barrier function and SCFA-mediated insulin signaling [34, 35]. This dysbiosis coincided with a reduction in hepatic GPX1 and SELENOW, selenoproteins essential for mitigating oxidative stress. Given that excessive mitochondrial reactive oxygen species generation is known to impair insulin signaling and promote glucose intolerance [36], the modest 11% improvement in early-phase glucose disposal following *R. torques* gavage in Se-deficient mice may be linked to the observed 1.6-fold partial restoration of hepatic GPX1. While *R. torques* appeared to act as a pathobiont in Se-adequate conditions by potentially reducing hepatic GPX1 and SELENOP, it may uniquely modulate Se utilization or inflammatory signaling in a deficient state, therefore providing a modest metabolic buffer. Furthermore, because it remains unknown whether *R. torques* expresses selenoproteins or utilizes Se, plausible competition between the bacterium and the host for Se in the gut is unclear. Altogether, the persistent depletion of *Roseburia/E. rectale* likely maintained a state of low-grade inflammation, which may explain why insulin resistance was not fully reversed despite partial restoration of antioxidant capacity [30].

This study has certain limitations, primarily the small pilot cohort (*n* = 4) utilized for this exploratory characterization of the under-studied R. *torques* strain. This constraint may limit the detection of subtle metabolic shifts and the generalizability of findings within highly variable microbiota-host interactions. Furthermore, mechanistic investigations, such as mucin content in intestinal epithelium, were not performed. Consequently, we explicitly state this limitation and advise interpreting these findings with caution as hypothesis-generating rather than definitive.

In summary, our results show that the administration of the *R. torques* ATCC BAA-2281 strain is associated with a transient attenuation of peak glucose levels in Se-deficient female mice, rather than a broad improvement in glucose tolerance, as the overall AUC and insulin sensitivity remained largely unaffected. Changes in five other bacterial taxa display patterns distinct from those observed with *A. muciniphila* oral gavage in male mice [16]. Further studies are needed to deepen our understanding and provide mechanistic insight into how *R. torques* influences Se status and type 2 diabetes in the host.

## Supplementary Information

Below is the link to the electronic supplementary material.

Supplementary Material 1

Supplementary Material 2

Supplementary Material 3

## Data Availability

No datasets were generated or analysed during the current study.

## Abbreviations

AKT: mouse thymoma viral protooncogene
GPX1: glutathione peroxidase 1
GPX3: glutathione peroxidase 3
Se: selenium
SELENOH: selenoprotein H
SELENOP: selenoprotein P
SELENOW: selenoprotein W
SCFA: short-chain fatty acid
