# Supplementary material for: Impact of Ruminococcus torques Administration on Glucose Tolerance and Hepatic Selenoprotein Expression in Selenium-deficient Mature Female Mice
Source: Biol Trace Elem Res. 2026 May 13;204(8):6223–32. doi: 10.1007/s12011-026-05106-5 (PMC13369740; doi:10.1007/s12011-026-05106-5)
Supplement: Supplementary file 1 — Supplementary Material 1 [file 12011_2026_5106_MOESM1_ESM.pdf]

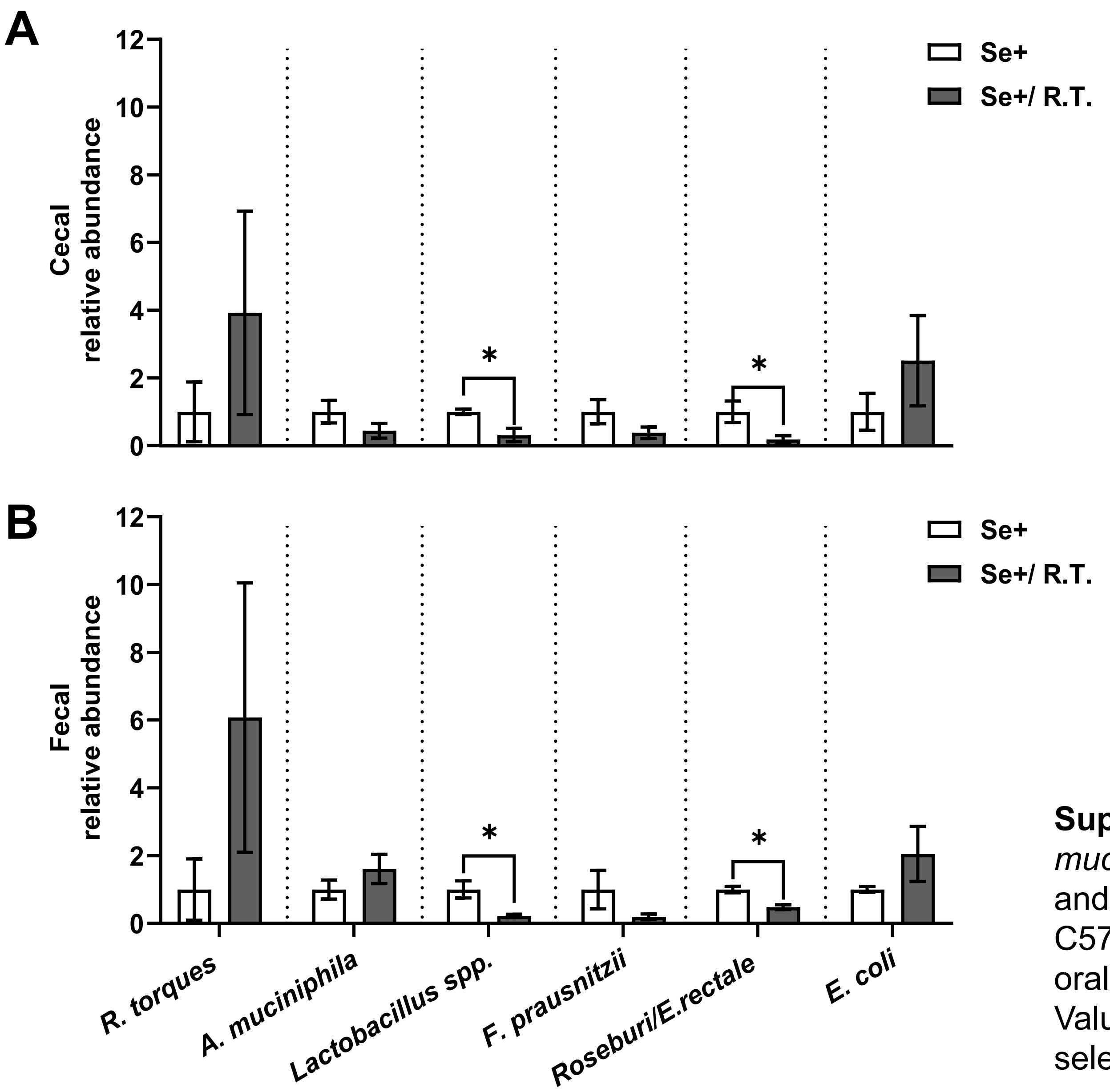

Supplemental Figure 1. Relative abundance of *R. torques*, *A. muciniphila*, *Lactobacillus spp*., *F. prausnitzii*, *Roseburia/E. rectale*, and *E. coli* in cecal (A) and fecal (B) samples from female C57BL/6J mice fed a Se-adequate diet with *R. torques* or mock oral gavage (See Figure 1A for detailed experimental design). Values are means ± SEMs (n = 4). **P* < 0.05. R.T., *R. torques*; Se+, selenium-adequate diet; Se−, selenium-deficient diet.

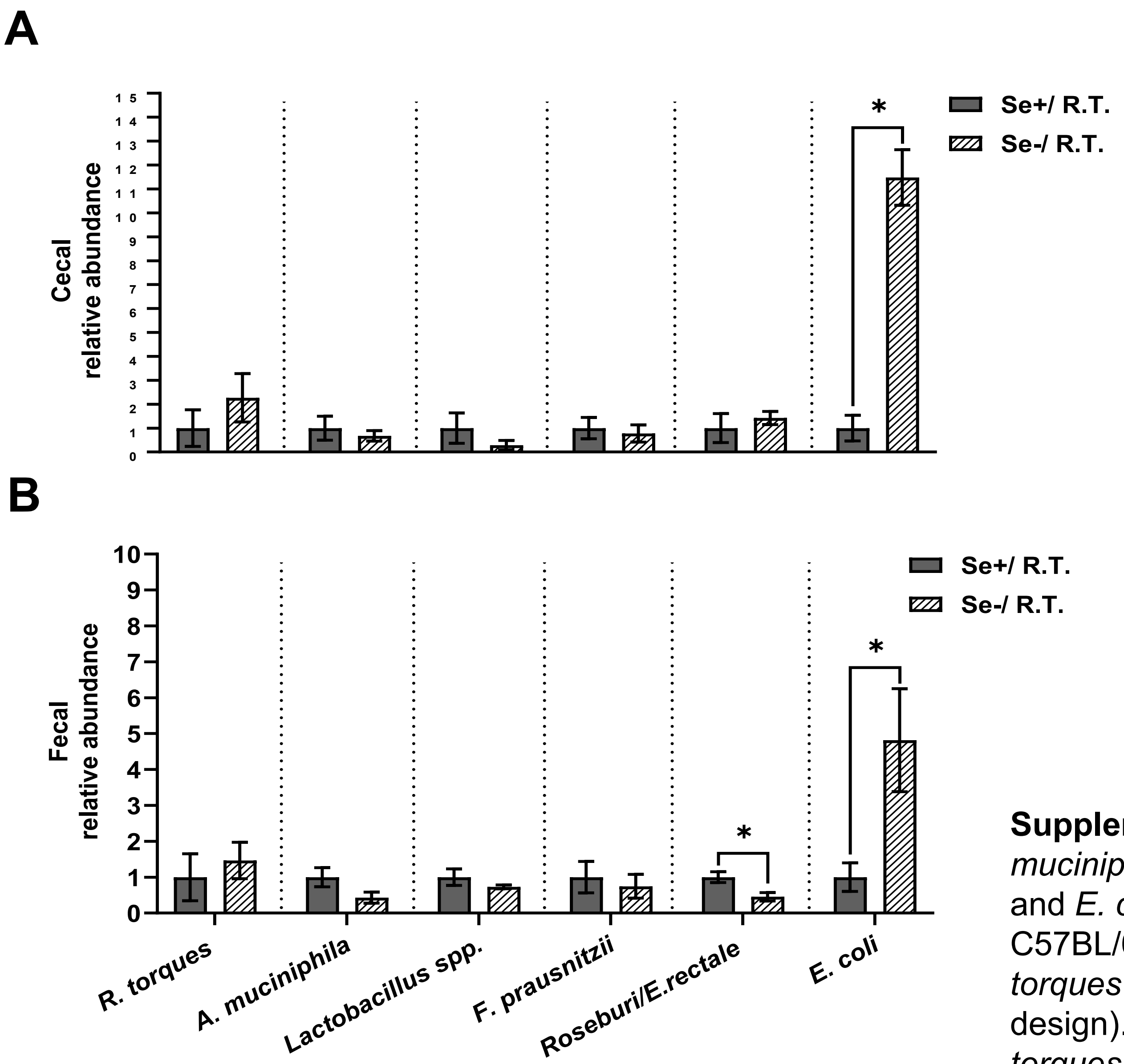

Supplemental Figure 2. Relative abundance of *R. torques*, *A. muciniphila*, *Lactobacillus spp*., *F. prausnitzii*, *Roseburia/E. rectale*, and *E. coli* in cecal (A) and fecal (B) samples from female C57BL/6J mice fed a Se-adequate or Se-deficient diet with *R. torques* oral gavage (See Figure 1A for detailed experimental design). Values are means ± SEMs (n = 4). **P* < 0.05. R.T., *R. torques*; Se+, selenium-adequate diet; Se−, selenium-deficient diet.
