# Supplementary material for: Impact of Ruminococcus torques Administration on Glucose Tolerance and Hepatic Selenoprotein Expression in Selenium-deficient Mature Female Mice
Source: Biol Trace Elem Res. 2026 May 13;204(8):6223–32. doi: 10.1007/s12011-026-05106-5 (PMC13369740; doi:10.1007/s12011-026-05106-5)

Supplemental Figure 3

Uncropped Figure 4 images

Muscle SELENOP

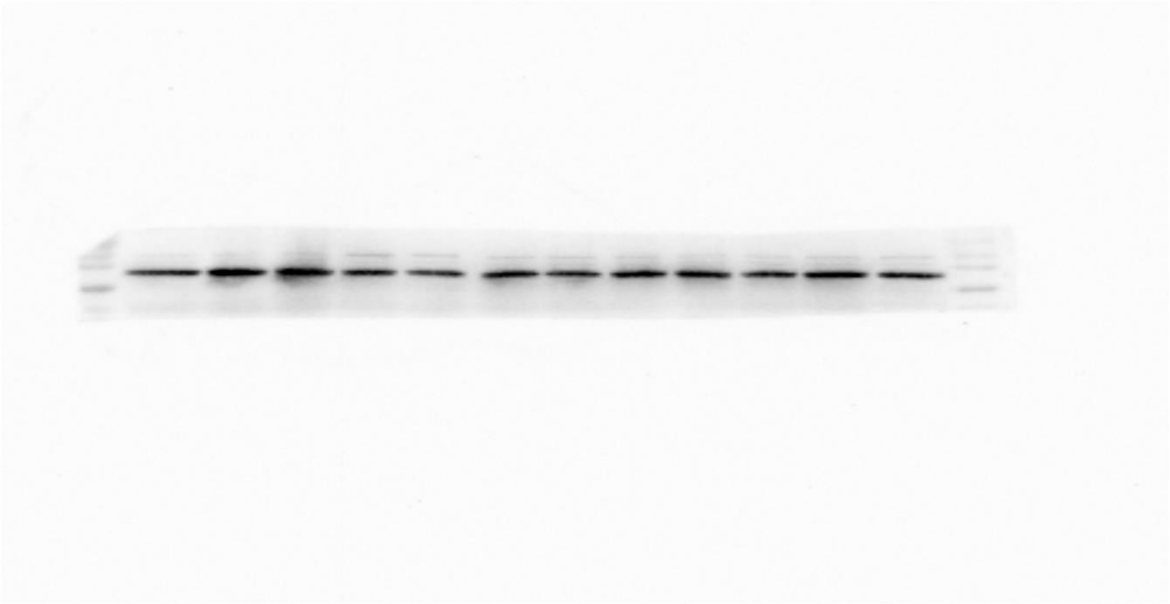

Muscle GPX1

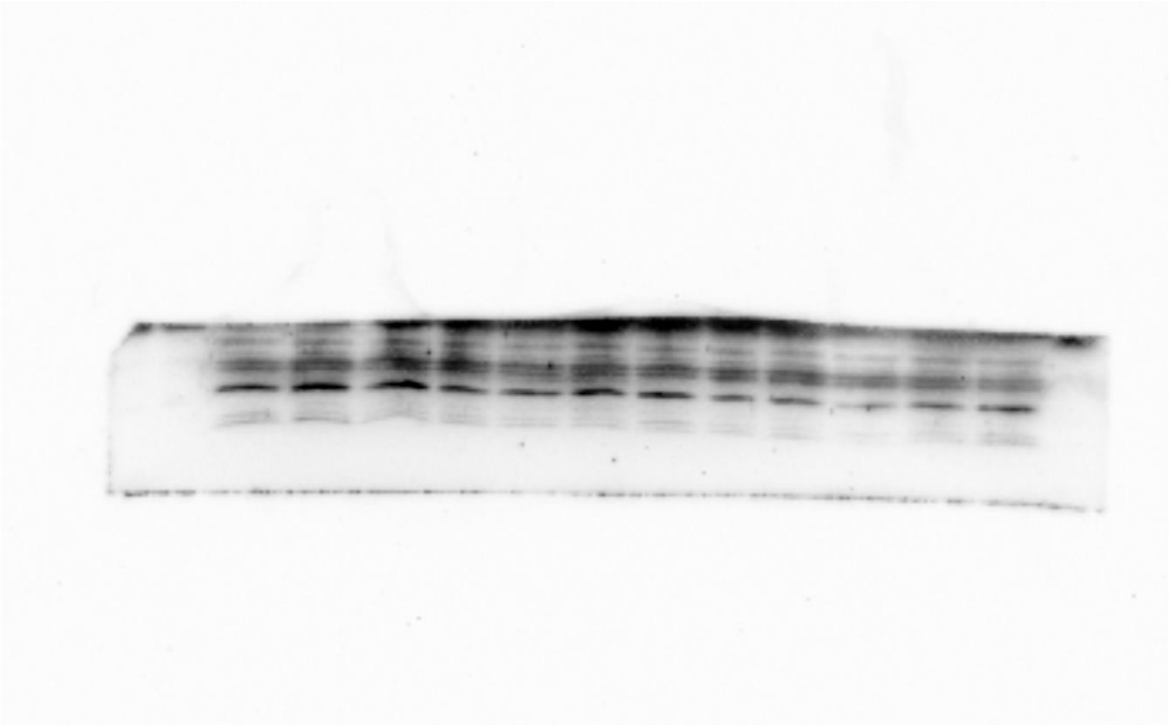

Muscle SELENOH

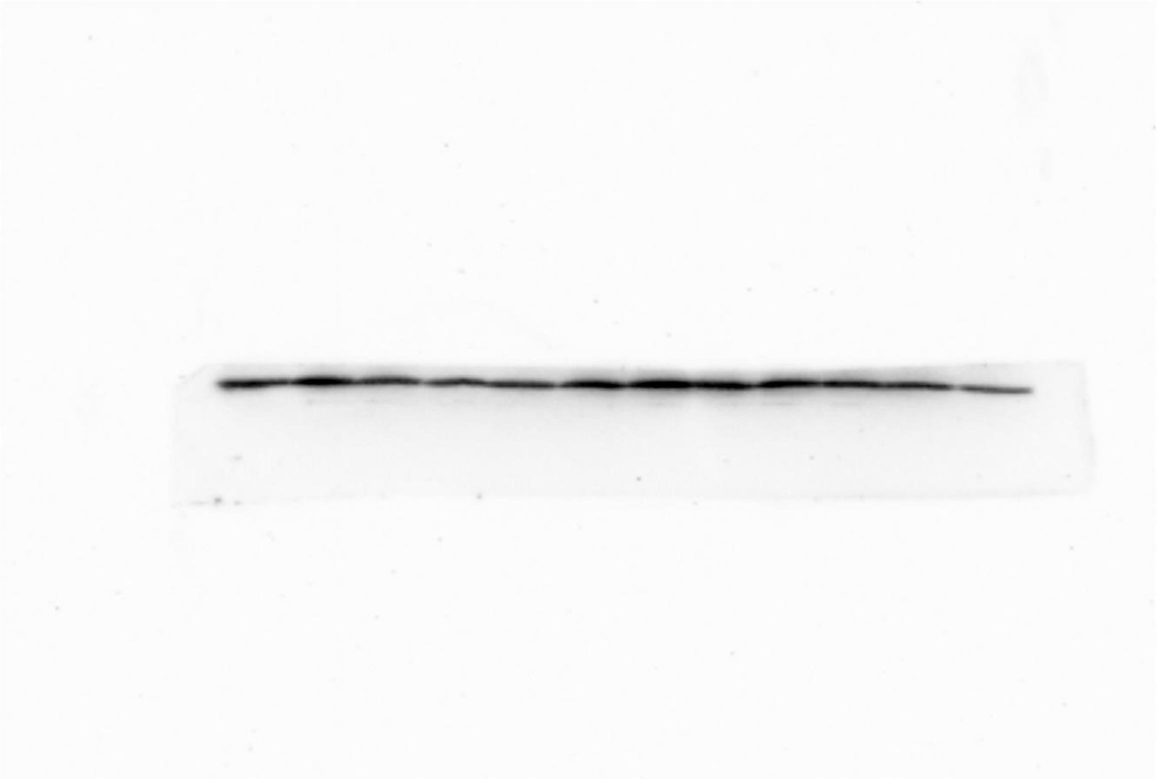

Muscle SELENOW

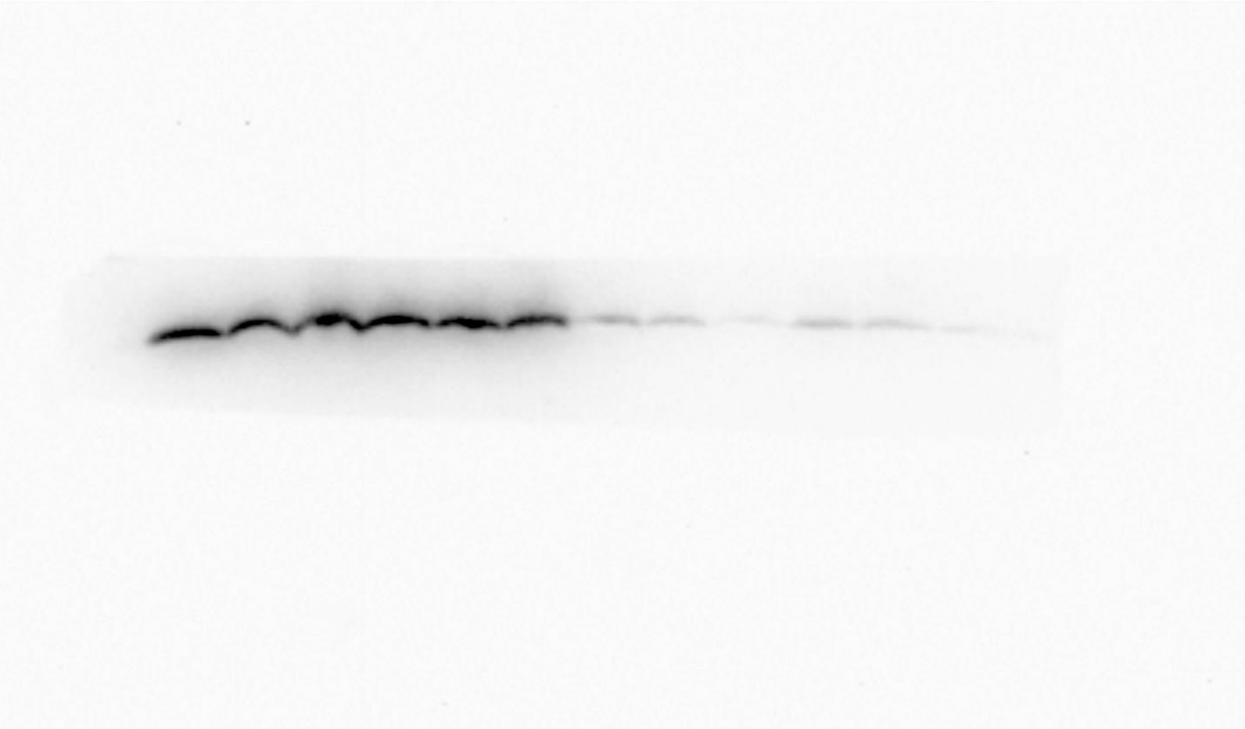

Muscle β-tubulin

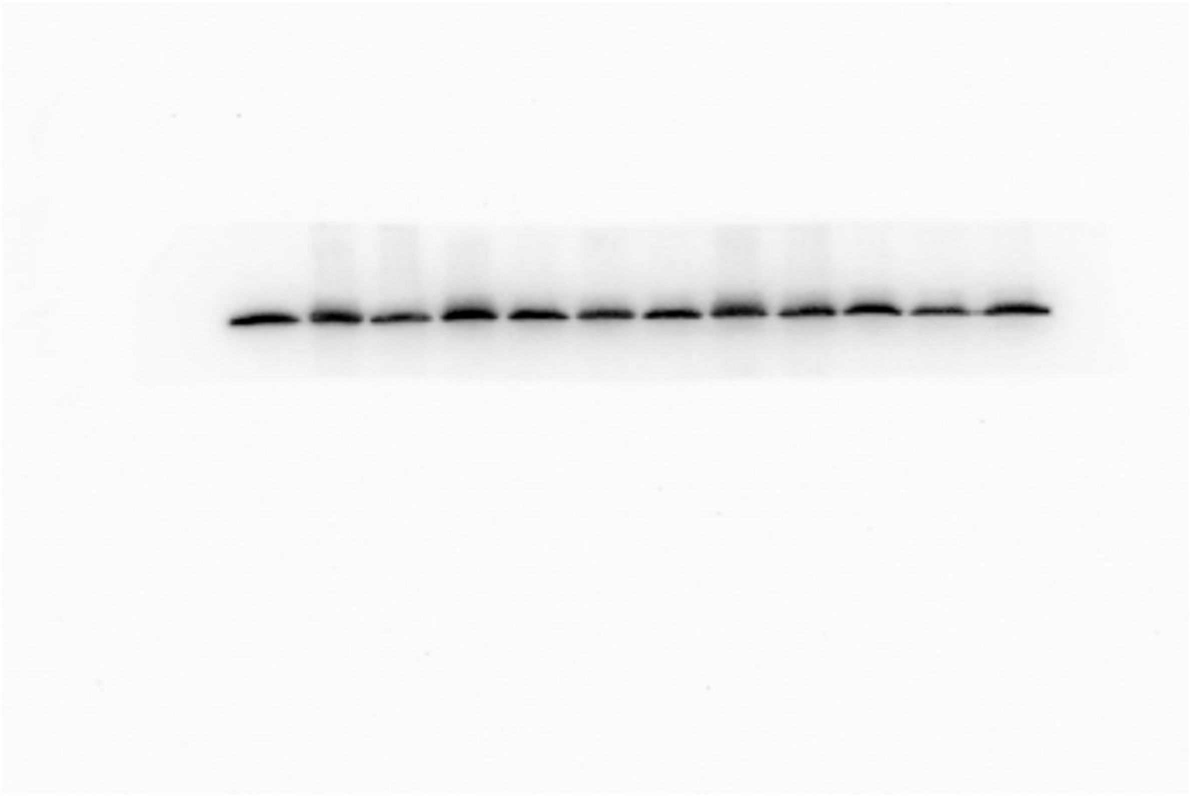

Liver SELENOP

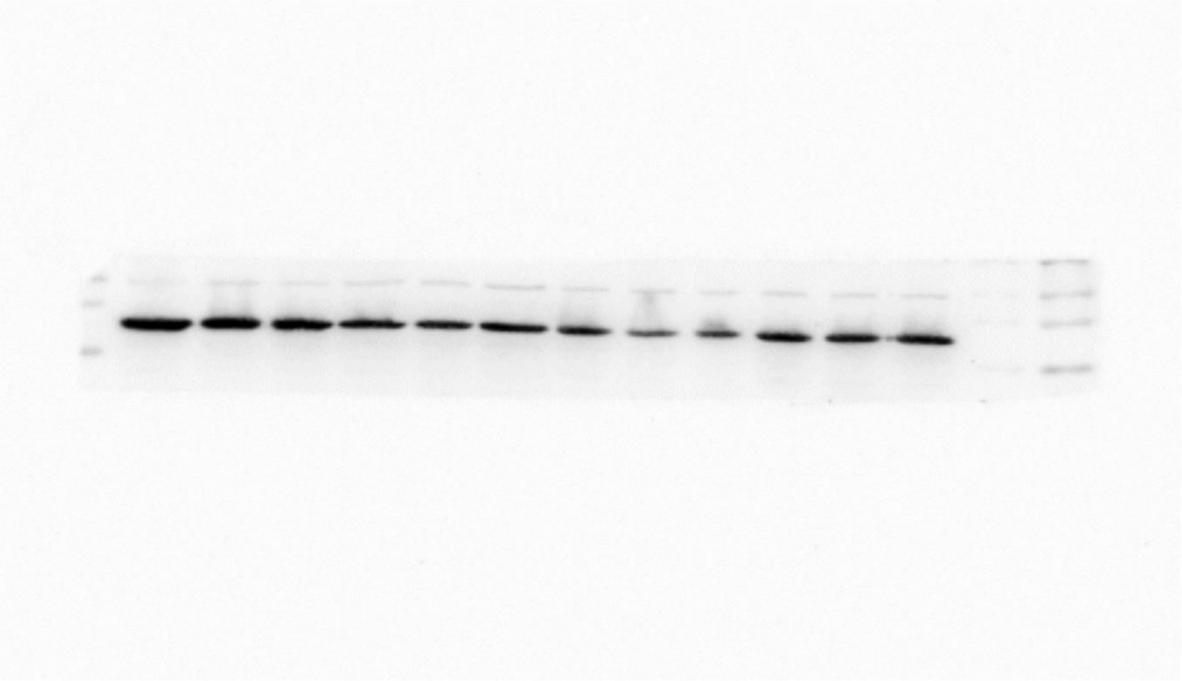

Liver GPX1

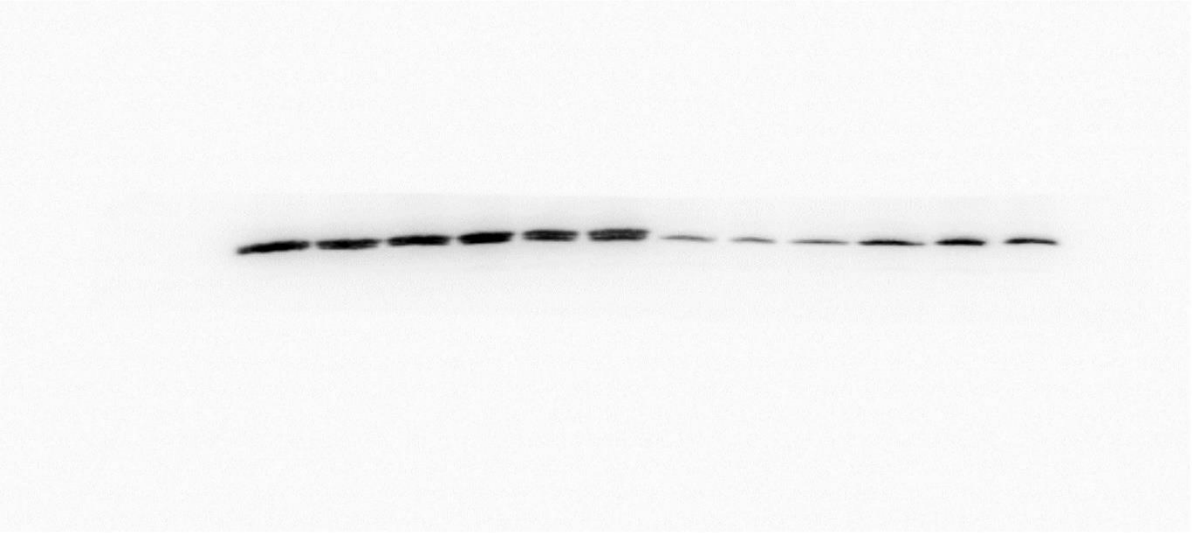

Liver SELENOH

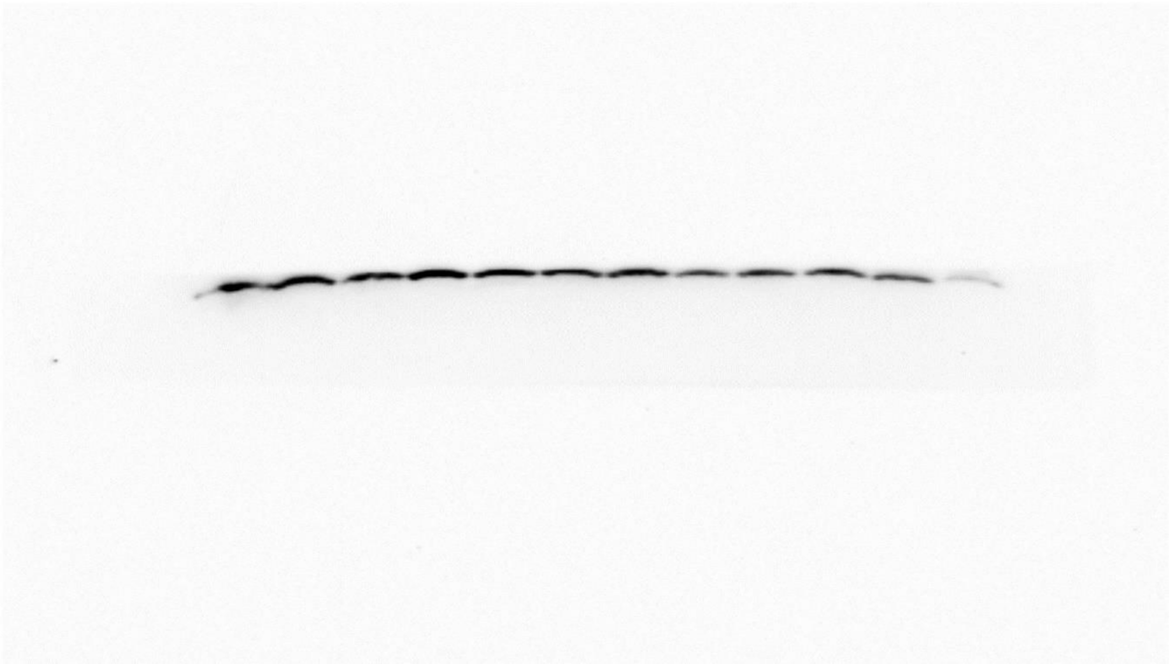

Liver SELENOW

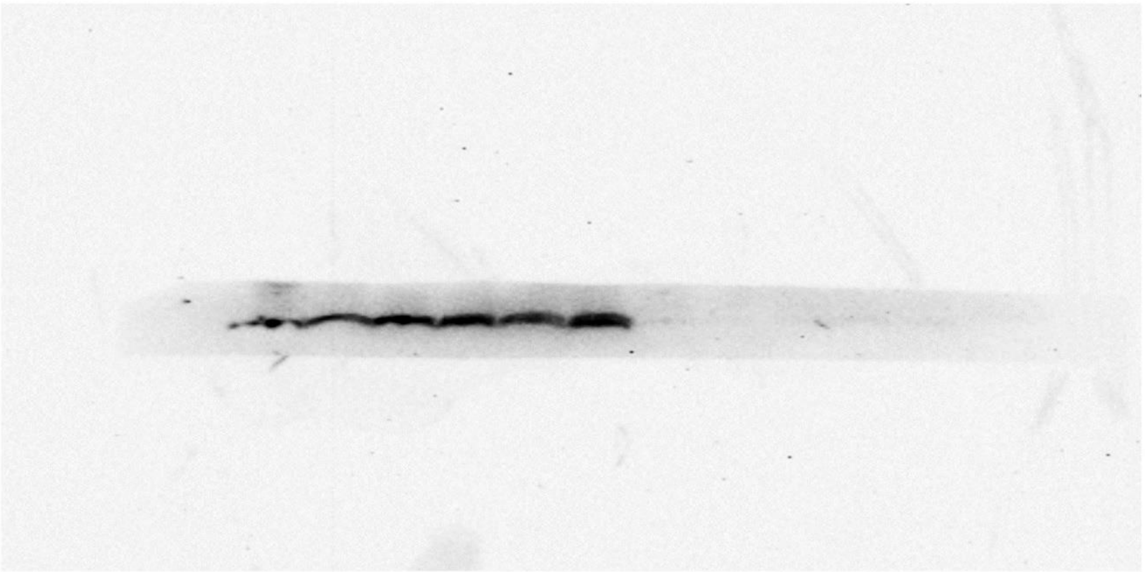

Liver β-tubulin

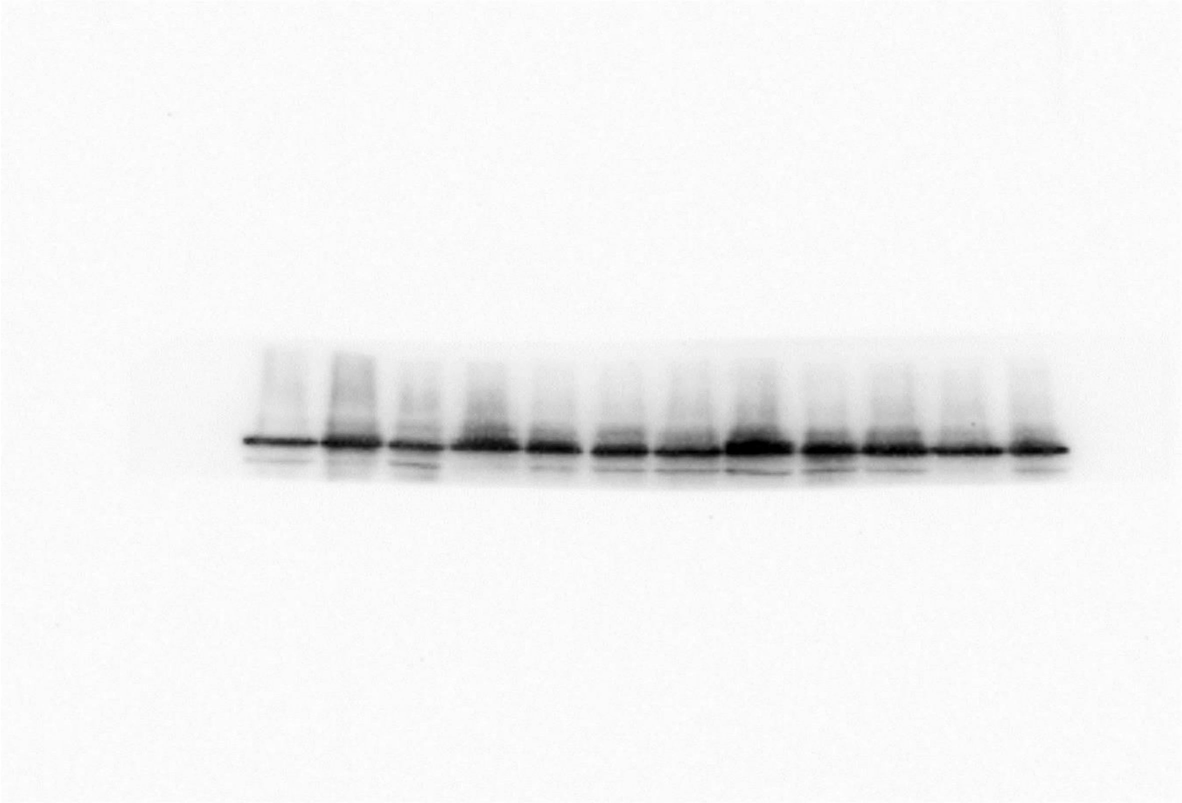

Supplement: Supplementary file 2 — Supplementary Material 2 [file 12011_2026_5106_MOESM2_ESM.pdf]
