# Supplementary material for: Impact of Ruminococcus torques Administration on Glucose Tolerance and Hepatic Selenoprotein Expression in Selenium-deficient Mature Female Mice
Source: Biol Trace Elem Res. 2026 May 13;204(8):6223–32. doi: 10.1007/s12011-026-05106-5 (PMC13369740; doi:10.1007/s12011-026-05106-5)
Supplement: Supplementary file 3 — Supplementary Material 3 [file 12011_2026_5106_MOESM3_ESM.pdf]

Supplemental Table 1. Primer sequences

Gene target	Sequence (5'-3')	References
Akkermansia muciniphila	F: 5'-CAGCACGTGAAGGTGGGGAC-3'<br>R: 5'-CCTTGCGGTTGGCTTCAGAT-3'	[1]
Escherichia coli	F: 5'-GGCCTTCGGGTTGTAAAGTA-3'<br>R: 5'-AGACTCAAGCTTGCCAGTATC-3'	[2]
Faecalibacterium prausnitzii	F: 5'-AGATGGCCTCGCGTCCGA-3'<br>R: 5'-CCGAAGACCTTCTTCCTCC-3'	[2]
Lactobacillus spp.	F: 5'-TGGAACAGRTGCTAATACCG-3'<br>R: 5'-GTCCATTGTGGAAGATTCCC-3'	[3]
Roseburia spp./ E. rectale	F: 5'-GCGGTRCGGCAAGTCTGA-3'<br>R: 5'-CCTCCGACACTCTAGTMCGAC-3'	[4]
Ruminococcus torques	F: 5'-TGCTTAACTGATCTTCTTCGGA-3'<br>R: 5'-CGGTATTAGCAGTCATTTCTG-3'	[5]
Universal 16S rRNA (V4 region)	F: 5'-GCCAGCAGCCGCGGTAA-3'<br>R: 5'-GACTACCAGGGTATCTAAT-3'	

Reference:

[1] M.C. Collado, M. Derrien, E. Isolauri, W.M. de Vos, S. Salminen, Intestinal integrity and Akkermansia muciniphila, a mucin-degrading member of the intestinal microbiota present in infants, adults, and the elderly, Applied and environmental microbiology 73(23) (2007) 7767-70.
[2] R.F. Wang, W.W. Cao, C.E. Cerniglia, Phylogenetic analysis of Fusobacterium prausnitzii based upon the 16S rRNA gene sequence and PCR confirmation, International journal of systematic bacteriology 46(1) (1996) 341-3.
[3] R. Byun, M.A. Nadkarni, K.L. Chhour, F.E. Martin, N.A. Jacques, N. Hunter, Quantitative analysis of diverse Lactobacillus species present in advanced dental caries, J Clin Microbiol 42(7) (2004) 3128-36.
[4] C. Ramirez-Farias, K. Slezak, Z. Fuller, A. Duncan, G. Holtrop, P. Louis, Effect of inulin on the human gut microbiota: stimulation of Bifidobacterium adolescentis and Faecalibacterium prausnitzii, The British journal of nutrition 101(4) (2009) 541-50.
[5] A. Kassinen, L. Krogius-Kurikka, H. Mäkivuokko, T. Rinttilä, L. Paulin, J. Corander, E. Malinen, J. Apajalahti, A. Palva, The fecal microbiota of irritable bowel syndrome patients differs significantly from that of healthy subjects, Gastroenterology 133(1) (2007) 24-33.

Supplemental Table 2. Antibodies used for immunoblotting[1]

Antibodies	Dilution	Catalog number	Company
Primary antibodies			
GPX1	1:2000	GTX116040	GeneTex, Irvine, CA
SELENOH	1:1000	ab151023	Abcam, Cambridge, UK
SELENOP	1:1000	GTX63138	GeneTex, Irvine, CA
SELENOW	1:1000	NBP1-49599	Novus Biologicals, Littleton, CO
β-tubulin	1:5000	sc-55529	Santa Cruz Biotechnology, Santa Cruz, CA
Albumin	1:5000	4929	Cell signaling technology, Boston, MA
Secondary antibodies			
Anti-mouse	1:5000	7076	Cell signaling technology, Boston, MA
Anti-rabbit	1:5000	7074	Cell signaling technology, Boston, MA

[1]Abbreviations used: GPX1, glutathione peroxidase-1; SELENOH, selenoprotein H; SELENOP, selenoprotein P; SELENOW, selenoprotein W.
